# "The Hospital" Nursing Mirror

**Published:** 1900-01-06

**Authors:** 


### "The Hospital" Nursing Mirror.

Being the Nursing Section of "The Hospital."

[Contributions for this Section of "The Hospital" should be addressed to the Editor, The Hospital, 28 & 29, Southampton Street, Strand, London, W.C., and should have the word "Nursing" plainly written in left-hand top corner of the envelope.]

### Notes on News from the Nursing World.

### THE NURSES OF NO. 5 GENERAL HOSPITAL FIELD FORCE, SOUTH AFRICA.

On Saturday the nurses of No. 5 General Hospital sailed in the "Moor" for the front, Superintendent M. Thomas, of the Army Nursing Service, in charge. Miss Thomas has been superintendent of the Cambridge Hospital at Aldershot for the past three years, and has had long and varied experience in the Army Nursing Service. She served in Egypt from 1882 to 1887, receiving the Royal Red Cross in recognition of her services. Sister A. B. Noble, of whose experience mention was made in a recent number of The Hospital "Nursing Mirror," and Sister Evelyn Kitching accompany her. They have both been doing duty at Aldershot since October. Miss Kitching was trained at St. Mary's Hospital, Paddington. She then became sister at Sir Patrick Dun's Hospital, Dublin, and after holding that post for two years, was Sister of the surgical ward at the National Hospital, Queen Square, London. In May last she was appointed assistant matron, and this post is being kept open for her until her return from South Africa.

### DUBLIN NURSES FOR THE FRONT.

A contingent of nurses from the City of Dublin Nursing Institution also left Southampton in the "Moor" on Saturday for the seat of the war. This institution was the first in the kingdom to subscribe to the fund for the relief of the widows and orphans, the response to the appeal of the lady superintendent, Mrs. Kildare Treacy, being so liberal that in five days she had received £14. It is said that nearly the whole of the large staff would gladly volunteer for service at the front, but it is a compliment to all of them that the Central British Red Cross Committee added four of their number to the Army Nursing Service Reserve. Within a week of their appointment three of them received orders to embark. They are all natives of Ireland, women of wide experience in their profession, and have for several years been attached to the City of Dublin Nursing Institution. The desire of the staff to nurse the wounded was first made known to the late Mr. W. J. de Courcy Wheeler, vice-chairman of the institution, just before his fatal illness in November, and he communicated it to the chairman, Lord Justice FitzGibbon. The latter received the valuable help of the Countess Cadogan, who personally interviewed each of the nurses at the institution. Her Excellency interested herself most kindly in their preparations, and wished them an earnest God-speed and safe return.

### THE TRAINING OF THE DUBLIN NURSES.

Miss Mary Talbot, of Westmeath, was trained in the South Infirmary, Cork; the City of Dublin Hospital; and Cork Street Fever Hospital, Dublin. She has served as a charge nurse in Belmullet Infirmary, in Banbridge Infirmary, in Mespil Private Hospital, and in the City of Dublin Hospital. She received the decoration of Serving Sister of the Hospital of St. John of Jerusalem for her arduous service during the typhus epidemic on the Island of Inniskea in 1895. Miss Sarah J. Callwell, of Dublin, holds the same decoration. She was trained in the City of Dublin Hospital; has served as a nurse in the Orthopædic Hospital, Dublin, and the City of Dublin Hospital, Mespil Hospital, and Dunfanagh, Castlebar, and Bellmullet Infirmaries. For a time she had sole charge of Kenmare Infirmary, and has had to deal with both typhus and typhoid epidemics, as well as with most critical surgical cases. Miss Mary Anna Davis, of Waterford, has seen nearly six years' service, and has held the position of nurse in Cork Street Fever Hospital, Roscrea Infirmary, Charlemont Street Hospital, Dublin, Mespil Hospital, and the City of Dublin Hospital, where she was trained. Miss Rosa Lawless, of Dublin, has had sole charge of Castlebar Fever Hospital during an outbreak of typhus, and has served in Lisburn Fever Hospital and in Mespil Hospital.

### A WESTMINSTER MATRON FOR THE FRONT.

Miss Jessie Southwell, Matron of the Westminster Training School and Nurses' Home, leaves for the seat of the war on Saturday, January 13th. Miss Southwell was trained at St. Bartholomew's, where she remained as staff nurse until December, 1894. She was next student at the Clapham School of Midwifery until August, 1895. From that date up to April, 1897, she was night superintendent and assistant matron of Chelsea Hospital for Women. She was then matron of the Royal Hospital for Children and Women at Bristol until October, 1898, and has since been matron of the Westminster Training School and Nurses' Home. Miss Southwell will carry with her to South Africa the good wishes of a large circle of friends.

### AN INSUFFICIENT NURSING STAFF ON THE "KILDONAN CASTLE."

We understand that, although no less than a hundred wounded soldiers were in charge of Dr. Wellbourne on the "Kildonan Castle," which arrived last week, there were only three nurses in attendance. These ladies did not belong to the Army Service or the Reserve, but were engaged at Cape Town. One of them was an Irish nurse, who rendered excellent service. Fortunately, the soldiers themselves did the best they could to make the work as light as possible for the nurses, but the number of the latter was obviously inadequate; and we hope that when Dr. Wellbourne, who left for South Africa again in the "Kildonan Castle" on Wednesday, brings back another cargo of the wounded in the same vessel he will be supplied with a larger staff.

### "TOO ENGLISH" FOR THE BOERS.

A Sheffield lady, who until recently was a nurse in the Johannesburg Hospital, writes from Fern Hill, Howick, *viâ* Pietermaritzburg, to her relatives in Yorkshire, under date of October 27th, 1899:—

"I had a letter, dated the 15th of September, sent on from

Johannesburg. It had been opened by the Boers. You will see by the papers that the English nurses and probationers were turned out of the hospital. About nine of these sailed from England with me, and the rest were English girl probationers, 40 in all. Six remained—Dutch and Colonial probationers—and about 20 of the Roman Catholic sisters. The doctor, a Frenchman, who had instructions from President Kruger to dismiss the nurses, insulted the nurses shamefully. He told them they were rebels, and untrustworthy, and that Miss Young's (the matron's) face was a d—— sight too English to have about the hospital."

This, it will be observed, is another version of the story already told in these pages of the expulsion of English nurses from the Johannesburg Hospital, but, according to the latest account, the language of the foreign doctor was even more offensive than was at first reported.

### GROSS MISMANAGEMENT AT ST. GEORGE'S INFIRMARY.

We draw attention to some grave charges made by a correspondent elsewhere respecting a London Poor Law Infirmary, which, on inquiry, we learn is St. George's Infirmary, Fulham Road. We are not only perfectly satisfied as to the veracity of our correspondent, but have been able to ascertain that there is a strong independent movement in favour of reform of the institution alluded to. We also learn that since our correspondent communicated with us things have become worse; but it seems that, having individually addressed a separate written protest to the committee, the nurses are hopeful respecting the redress of their grievances. We sincerely trust that their expectations will be realised, and we are very glad to hear that the matron has strongly appealed to the committee for more nurses, though so far her request has, apparently, been disregarded. It will be observed that a night nurse has two floors to attend to. What this means is shown by an incident which occurred some time ago. A nurse was attending to a patient in a violent fit in the lower ward and at the same moment a woman in the upper ward was seized with a similar attack, followed, as usual in her case, by a kind of frenzy. As there is no bell connecting the wards, the nurse could not be summoned, though, in any event, the other case demanded all her attention. But in the absence of a nurse in the upper ward the woman in a fit held a pillow over the face of a very old and feeble patient, and it required the united efforts of several of the other inmates to rescue the poor creature, who was ill for days afterwards from the shock. The urgent need of a communicating bell, which is not an article of luxury; the inadequacy of the patients' and nurses' food; the deplorable arrangements of the wards; and the failure to ensure dry clothes for the nurses, are matters of the greatest importance, requiring the immediate attention of the authorities. Having taken up the question we do not intend to allow it to drop until the conditions of nursing at St. George's Infirmary have been thoroughly reformed. There is not, in this instance, even the poor excuse of want of funds.

### THE SCARCITY OF NURSES IN THE CALCUTTA HOSPITALS.

The last report on the medical institutions of Calcutta is satisfactory as to the quality of nursing. The Principal of the Medical College, speaking generally, states that "the nursing was good." But it is unsatisfactory as to quantity. At the Ezra Hospital there is only one nurse, though two "at least" are required. There are "not enough nurses" at the Eye Hospital, and, in respect to the Howrah General Hospital, the Superintendent says that, as there are but two nurses for European wards, and one for the native female ward, and they have to do all the work day and night, "nursing in the strictest sense of the term cannot be performed." Yet again, while the nursing in the Sambhu Nath Pandit and Campbell Hospitals is also favourably mentioned by the superintendents, it is described as "capable of improvement by adding to the staff." We are therefore glad to learn that the question of improving the nursing arrangements in all the Calcutta medical institutions is under consideration, with a view to their amendment by the Government of India.

### THE TAUNTON NURSES.

Last week the annual distribution of the prizes and certificates won by the nurses at the Taunton and Somerset Hospital in their elementary anatomy and physiology examinations took place at the Nursing Institute. The prize and certificate for anatomy were won by Nurse A. Crouch, and certificates by Nurses Stella Rust, E. Westcott, and L. Rule. For physiology the prize and certificate were won by Nurse Rust; while Nurses L. Rule, Fairhurst, and Bloxham received certificates. Dr. Macdonald said it was very pleasing to those who had anything to do with the hospital to see how well the nurses did in that work every year, because it was not the ordinary work of the hospital, but additional work, and it showed the great amount of interest they took in their work that they should go in for these classes and take prizes. They must not suppose that those who had gained certificates were the only ones who went in for it. All the nurses took part, but, of course, they could not all win prizes. He hoped that those who had been unsuccessful this year would win certificates next year. Other speeches followed, and the company subsequently visited the wards, and took part in the usual Christmas festivities.

### THE NEW NURSES' HOME AT ABERDEEN.

The handsome new buildings erected for the use of the nurses of the City Hospital at Aberdeen were opened on Boxing Day in the presence of a large company. The cost of the important extension has been about £5,000, and the event is the more a matter for congratulation because it marks the completion of the entire hospital. At the interesting opening ceremony the speakers dwelt upon the necessity of possessing an efficient nursing staff, and it was maintained that nurses of a superior class could not be obtained unless they were adequately remunerated for their services, and provided with proper accommodation. Many compliments were paid to the matron, Miss Frater; and a feature of the proceedings was a dance, in which the nurses and a number of invited guests took part. The former expressed themselves highly delighted with their new and admirably arranged quarters.

### AN IRISHMAN'S IDEA OF NURSING FEVER.

A nurse in charge of an Irish parochial hospital sends us the following:—

"Some few weeks ago I was startled by a loud knocking at the door. On going to see what was wrong I met a man who was evidently in a very excited state. He said, 'Are you the woman who looks after the feverish people?' On my replying 'Yes!' he rejoined, 'I'm glad to hear it, ma'am, for, God bless you, you seem to have a good honest face.' I gently inquired what his business was. 'Well, ma'am, I want you to go at wanst (once) and air a bed and bedclothes, for I have a son very sick at home, and the doctor says it's the faver his caught, and that he must come in here. And now, ma'am, I want to talk to you about my son. He had a good, clane feather bed at home, and every comfort under the sun, and for nursing, there is nothing to equal the nursing he got. Herself (meaning his wife) and myself never tired of doing everything for him—we even got up a few times in the night to give him a drink. Of course we can't expect you to nurse him half as well as we did, but still I know by your honest face you will do your best. I often heard how those women that look after the faver people are cruel and unkind, and that they starve the sick. Now, like a good girl, give my boy plenty to eat, plenty to drink, and don't forget to get up in the night a few times to see how he is going on. I won't let my son in for four hours, to give you plenty of time to air the bed and bedclothes, and to prepare for him in every way. When he is better, herself and myself won't forget you.' As I saw the poor man's ignorance and simplicity, I thanked him for his kindness, and told him I would indeed do my best for his boy. The patient came after the specified time, and as he was an extremely bad case—temperature over 106 a few times, but is now nearly fit to be sent back to his beloved parents—I think I can safely say I did do my best for him, especially as there is no other nurse here, and I have only a servant to help me."

### THE NURSES' NEEDLEWORK GUILD.

The third annual report of the Nurses' Needlework Guild, which was established to assist needy hospital patients with gifts of warm, useful new clothes, shows that progress continues to be made. Twenty-two new members were enrolled during the 12 months, and 490 garments were sent in for the annual show, all of which were most useful and well made. This is 47 in excess of the number in 1898, and they were sent in parcels to the following hospitals: The London, Guy's, St. Thomas's, St. Mary's, the Royal Free, the East London, University, the Brompton Consumption, the Metropolitan, Nazareth House, and Clapham Maternity Hospital.

### NURSES AT THE LIVINGSTONE EXHIBITION.

On Wednesday afternoon a number of nurses were present at the Livingstone Exhibition in St. Martin's Town Hall, organised under the auspices of Livingstone College. The interest taken just now in anything African was manifested on the occasion, and the various kinds of devices for making the work of the explorer easy, and for reducing to a minimum the transport of a military expedition, attracted special attention. The loan collection of Livingstone relics—his sextant, Bible, journals, pistols, surgical instruments, autograph maps, and other objects connected with his life and work—was a very popular feature of an admirably arranged show.

### NURSING INSTITUTIONS IN SWITZERLAND.

A correspondent from Lausanne sends us the following information about a nursing institution of which no mention was made in an article published some time ago in these pages on "Nursing Institutions in Switzerland." It is called "La Source." Our correspondent says, "The training home occupies a house in the Chemin Vinet, in the immediate outskirts of Lausanne, and was founded in 1859 by Monsieur and Madame De Gasparin, as the 'Ecole Evangélique Indépendante de Gardemalades.' Their generosity continued to provide for its support during their lives, but in 1891 a change was made. Since that date it has been under the management of Dr. Krafft, and now consists of two institutions, combined under one roof, namely, the training school and the private hospital of Dr. Krafft. The pupils have in this manner the advantage of gaining practical, as well as theoretical, instruction. The course lasts for three years, of which eight months are passed in the home, and the remainder of the time in some hospital, or in private nursing. During their training the nurses also carry out district nursing in the town of Lausanne, some 7,000 visits being yearly paid in this way. At the end of the training a certificate is granted, after which the nurses are free to take up any work that may offer itself. The nurses are of two classes—those who reside in the home, to whom board and instruction are given free, and those who only attend the courses provided; in the case of the latter payment is expected. The number of nurses who have been trained since the commencement amounts to 800, and these may be found now scattered throughout the world."

### KINDNESS TO A SICK NURSE.

The nurses of the Queen Victoria Nursing Institution, Wolverhampton, have shown their sympathy to Nurse Evans, one of the staff, who has been ill since April, by collecting amongst themselves the sum of £2 12s. The committee, too, have also generously thought of her. For the last eight months Nurse Evans has been in the General Hospital, Wolverhampton, where she has received every care and attention from the matron and nurses, and is now convalescent and looking forward to getting a change of air.

### HE LEFT HIS MARK BEHIND HIM.

"She was an Irish girl, of 18," writes a nurse, "and occupied the corner bed, screened round, as she was a 'special' case, Cæsarian section, and had had a bad time of it. The baby had only survived 24 hours—a huge thing of 14 pounds. To-day her eyes shone, for when her boyish husband called to ask for her, as he did twice daily, he was to be invited in to see her. 'Shure, nurse,' she whispered to me, 'he must clane himself first; he is in the engine room, and gets so black.' 'Oh! never mind, only take care of the sheet,' I said, and presently I put him behind the screen, after searching him for forbidden eatables, &c. It was most amusing to see her when he had gone. She lay serenely happy, with her face and her mouth absolutely grimy! and when I suggested I should have to use some soap and water, she blushed most charmingly."

### SHORT ITEMS.

The course of instruction in midwifery at the Midwives' Institute commences this month. A fee of three guineas is charged for the course, and arrangements can be made for the practical work.—At the annual meeting of the Dorchester District Nursing Association the other day, a proposal to engage a second nurse was discussed, and the matter was ultimately referred to the committee, with every prospect of a satisfactory arrangement.—The nurses of the Blackheath Nursing Institution presented the Lady Superintendent, Miss Duncan, on Christmas Day, with a Sutherland tea table and handsome pair of candlesticks.—The usual entertainment for the nursing staff of St Thomas's Hospital and their friends will be given on Friday evening, January 26th. The "musical conversazione" last year was so successful that it is proposed to repeat it in place of the usual concert.

### Lectures to Nurses.

### THE NORMAL PUERPERIUM; CARE OF THE MOTHER AND CHILD.

By ROBERT JARDINE, M.D., &c., Physician to the Glasgow Maternity Hospital.

THE puerperium lasts from the end of the third stage of labour until the organs of generation have returned to their normal condition. This takes from six to eight weeks. The unimpregnated uterus is about three inches long and weighs about an ounce. Immediately after labour it is about seven inches long and weighs about two pounds. This large amount of tissue has to be absorbed and got rid of by the system. This process is known as involution.

Immediately after labour the fundus of the uterus lies slightly below the umbilicus. To be exact the position of the fundus should be measured from the top of the symphysis, but as the umbilicus is the landmark usually selected, we shall adopt it. During the first day the fundus usually rises slightly, and it may come a little above the umbilicus. After that it should fall steadily at about the rate of an inch a day, until by the tenth day it is at the brim of the pelvis. The involution now becomes slower, but in six or eight weeks from the end of labour the uterus should have returned to its normal size. Suckling tends to hasten involution, while a septic condition or anything retained in the uterus will retard the process.

LOCHIAL DISCHARGE.—Considerable discharge comes from the vagina during the first three weeks of the puerperium. It is red for the first three days or so (lochia rubra), then for three or four days it is watery or serous (lochia serosa), and after that it is whitish (lochia alba). It gradually decreases in amount. It has a peculiar heavy odour, but should not have a fœtid smell. As soon as it is exposed to air it becomes septic, hence the great necessity of receiving it into aseptic or antiseptic napkins, which, when soiled, should be removed and burned. For the same reason the external parts must be kept clean.

CARE OF THE MOTHER.—A nurse's responsibilities are very great. If she neglects her duties and allows her patient to become septic the result may be very serious. She must carry out the doctor's instructions implicitly, and if the case is one of her own she must try to have her own orders carried out as fully as possible. The room must be kept at a temperature of from 60 to 65 deg. Fahr., and it should be well ventilated. There should be a fire in it. When it is dusted, this should be done with a duster dampened with Sanitas or some other antiseptic. No soiled things should be kept in it. The fewer visitors allowed to see the patient, the better. After the mother has rested a few hours and has had some nourishment the child may be put to the breasts. The nipples should be first washed and drawn out.

CLEANSING THE PATIENT AND CHANGING THE DIAPERS.—During the first three days this should be done every four or six hours according to the amount of discharge coming. The soiled diaper should be removed and burned, and the external genitals gently cleansed with lysol solution before the fresh diaper is applied. The binder and drawsheet should be changed when soiled. They should be put into an antiseptic solution at once, and should be washed and boiled before being used again. When cleansing the patient or doing anything about the breasts the nurse must sterilise her hands, and she must remember to have them warm. Five or six hours after labour the patient should be encouraged to make water. The external genitals should first be thoroughly cleansed with a warm antiseptic solution, and some warm water should be left in the utensil. The vagina will be thoroughly drained in this way, and the risk of sepsis lessened. If she cannot pass urine by ten hours after labour a catheter must be passed with due aseptic precautions. The parts should be cleansed and the catheter rendered aseptic. Pass it by sight and not by touch. The use of a dirty catheter may set up cystitis, and cause the woman months of suffering. A midwife should visit her patient within twelve hours of labour, and she should always inquire if urine has been passed. If it has not she must get her patient to try to pass it, and if she cannot the catheter should be used. She must thoroughly cleanse her patient, change the diaper, tighten or change the binder, and clear away all soiled things from under her.

The temperature and pulse should be taken night and morning, or oftener if necessary. Both should remain about normal, but there may be a slight rise of both on the third day, when the breasts become engorged. The temperature may rise to about 100·5 deg., and the pulse to 100 or so, without there being any serious complication; but if they rise higher than that always be suspicious of sepsis. When a sudden rise of several degrees occurs the doctor should be notified at once, but do not alarm your patient. The pulse is really a more valuable indicator of the patient's condition than the temperature. Between 60 and 90 may be taken as a safe range.

THE DIET.—The patient will not care for much food for the first two or three days, but she will probably be thirsty. Until the bowels are moved her food should be chiefly liquid, such as tea and toast, milk puddings, gruel, soups, beef-tea, milk and soda, and porridge in the morning if she cares for it. If she is not to nurse, the amount of fluid should be restricted. After her bowels are well cleared more solid food can be given, such as eggs, fish, chop, chicken, or rabbit, and in a few days ordinary plain food. If she is nursing she must take a good deal of farinaceous food and plenty of milk. Stimulants should only be given when ordered by the doctor.

EVACUATION OF THE BOWELS.—The bowels are usually constipated, and they must be cleared out forty-eight hours after labour. Castor oil is by far the best purgative to use. A tablespoonful usually suffices. If the patient is not to nurse, a saline should be used, such as Epsom salts, a Seidlitz powder, or one of the mineral waters. An enema of soap and water may be required. Sometimes inflamed and prolapsed piles give trouble. They should be well fomented. During the remainder of the puerperium a daily evacuation of the bowels should be secured. If the patient is strong enough she should be allowed to sit on the utensil while the bowels are moving. This allows of free drainage of the uterus and vagina.

AFTER PAINS.—Nearly every multiparous woman suffers from these. When they occur in primiparous cases there is usually something retained in the uterus. It may be a clot, piece of placenta, or membrane. The pains last from twelve to forty-eight hours, but usually cease after the bowels have been cleared out. Suckling increases them. If they are very severe a dose of opium, fifteen to twenty drops of laudanum, or a quarter-grain morphia suppository may be given, and will usually relieve the pain.

QUIETNESS FOR THE PATIENT.—This is of the utmost importance. During the first week only one or two visitors should be allowed. The nurse must strictly enforce the doctor's orders. After the third day, if the patient is strong enough, she should have her shoulders raised slightly during the time she is taking her meals. About the seventh day, if she is able, have her lifted out of bed on to a couch or easy chair while you are making her bed. After a few days, she may be allowed to sit up for a short time. By the end of the second week she may move about the room a little. During the third week she may leave her room, but she should still lie down and rest a good deal. In summer she may go out for a drive or short walk by the end of the third week. She must not exert herself for at least two months, until the uterus has returned to its normal size. Among the working-classes so much care is impossible. They begin work far too soon, and that is the reason why so many of them suffer from falling of the womb.

### Mismanagement at a London Poor Law Infirmary.

GRAVE CHARGES BY A CORRESPONDENT.

There are a few facts connected with a London infirmary with which I am acquainted which seem to call for attention. Each ward, generally containing 28 beds, has one nurse to attend to it, except when there is an occasional probationer to assist her. There is, however, unusual pressure on space at the present moment, and the number of beds has been brought up to 36 in most of the wards, but the nurse is still single-handed. At night a nurse has always to undertake two wards on different floors, and you will easily understand how considerable are the strain and anxiety involved. While the nurse is engaged in the upper ward she is nervously apprehensive of what may be taking place in the lower one, and *vice versa*, there being no connecting bell to call her in case of need. There are often several dying patients in both (four phthisical cases died in one ward within a week quite lately), and sometimes there are half a dozen lunatics, who complicate matters amazingly.

The Patients' Food.

The quality of the patients' food is, I believe, excellent, but the quantity is surely inadequate. In many of our general hospitals five meals a day are customary, viz. : Breakfast, five a.m. ; lunch (beef tea, &c.), nine a.m. ; dinner, one p.m.; tea, five p.m. ; supper, eight p.m.

If that state of luxury be compared with the following, the difference is easily appreciated : - Breakfast (four ounces bread, small piece butter, mug of tea), seven a.m. ; dinner, one p.m. ; tea (same as breakfast), five p.m. Dinner consists of one of three diets as follows :—Meat and potatoes every day for a week except on Wednesday ; fish and potatoes every day for a week except on Wednesday ; pudding every day for a week except on Wednesday. The two former leave nothing to be desired, but pudding, however excellent, is scarcely nutritious enough to be the single viand at the only solid meal allowed in the twenty-four hours. The most serious cases on special diet are allowed a pint of warm milk daily, usually given about eleven a.m., and a pint of arrowroot about six p.m. ; besides an egg at breakfast and at tea in addition to the usual portion of bread. But when one reflects that for the majority there is a period of more than fourteen hours (five p.m. to seven a.m.) between the two least substantial meals, it can scarcely be expected that the sick should rally as rapidly as would be the case in an institution where nourishment is provided more frequently. I make a rule of never listening to complaints from patients, but many have remarked incidentally how hungry they are when bedtime comes.

I tried on one occasion to interest a well-known member of a vestry in the matter, but he said that no doubt the paupers got far more than they deserved, and that it doesn't do to make that class of person too comfortable.

The Need of Separate Wards.

A very serious mistake is made in not arranging separate wards for children. I speak more particularly about the male wards, which contain boys of all ages, though it is equally a lamentable thing to have young girls ten years of age and upwards in constant daily companionship (often for months at a time) with fallen women of the worst possible type. In the male ward the better kind of men have spoken about the exceeding wrong of young boys being among them. One, an old policeman, said to me recently that though his work in the force had taken him in past days to the worst slums and purlieus of London, he had never in all his experience heard such utter vileness of conversation as since he had been a patient there. Many of the unfortunates are literally taken out of the gutter. There are match-sellers, boot-blacks, tramps, loafers ; one man lately come tells me that he has "earned his living" ever since he can remember by "'anging outside pot 'ouses and runnin' herrands for 'apence." Of course there is a certain proportion of the decent labouring class among them, but can anyone delude himself into thinking it an atmosphere fit for boys of twelve, who are often the respectably brought up children of artisans. The children's maladies are seldom serious ; tinea is the usual complaint.

An Appeal to the Committee.

I understand that the undesirability of mixing children and adults was represented to the committee some time ago, but without result. Apart from the ruin of minds and morals that must inevitably ensue, there is another side of the question that ought not to be lost sight of—the extreme suffering it entails upon the dying. Three out of those four men who died in one week in the ward I mentioned spoke most piteously to me of the torture caused them by the noisiness of the boys, of whom there were then three among them. There is a little lad of five there now whose unceasing occupation is turning head over heels on his bed, or even taking flying leaps therefrom and hitting his neighbour (who is suffering from phthisis) on the head, knowing himself to be safe from pursuit as the man is far too ill to retaliate. As the nurse is often engaged in the ward kitchen there is very little check on these vagaries. In the bed on the other side of the child is an elderly man painfully recovering from pneumonia and pleurisy, and in constant agony. He was scarcely expected to live through last week, and that he should have contrived to rally with that little fiend in the next bed is nothing short of a miracle.

The Nurses Obliged to Wear Damp Clothes.

The nurses are not too luxuriously provided for. One of the wards is apportioned to them for sleeping quarters ; curtains divide it into cubicles containing two beds apiece. However, a plot of ground has been secured on which a nurses' home is to be erected. A month or two back I noticed that a usually rosy-cheeked nurse looked very white and ill. She told me that for several days past she had been suffering from acute rheumatism in every limb, and was scarcely able to get about. I should have passed by with the usual expression of sympathy had she not added that a good many of the staff were suffering similarly. This seemed strange, so I asked if she could account for it in any way. "Oh, yes," she replied, "it is because our clothes come home from the wash so wet, and our galatea dresses being very thick keep damp ever so long" (when worn, *bien entendu*). "Why do you not air them?" "Well, we have had no place to do so in, but after this week I think we shall be able to manage it, as the hot pipes are going to be put in use for the winter." I inquired whether she wore flannel next to the skin, and was told that she had done so ever since she had had a severe attack of rheumatic fever before coming to the infirmary, but that the flannel came from the laundry as damp as everything else.

The matron is a most excellent organiser and a thoroughly capable woman, but unfortunately the nurses are without exception too nervous to go frankly to her with their difficulties, yet that she has their welfare at heart is evident in many ways. She lately deprived herself of the much-needed afternoon off duty in order to check the clothes sent a second time from the laundry, to which she had returned them to be re-dried on discovering that they were disgracefully damp. Unfortunately the carelessness was not permanently prevented, and at present the nurses are surreptitiously dosing themselves with salicylic, instead of sensibly asking permission to see the doctor, and complaining to their head of the laundress' shortcomings.

THE NURSES' MEALS AND HOURS OF DUTY.

The nurses' meals, like the patients', seem to be inadequate, the last in the day being so many hours before bedtime that most of the nurses supplement it with small extras, or go hungry to bed.

The night nurses are called at a quarter to four or four p.m. Their hours of duty are unusually long—twenty minutes past seven p.m. to twenty minutes past seven a.m., when they go to bed. Since the recent granting of a church pass—the first accorded them in 22 years—they rise at a quarter-past ten on Sundays in time to dress for service, and on their return at a quarter-past twelve again retire to rest. Unluckily, the matron, has decided to forbid them to remain in bed after the usual hour for rising at a quarter to four, though it is difficult to see why they should not be allowed to make up for the disturbed morning sleep in the hours that are spent in recreation on other days, *i.e.*, from half-past four to half-past six. If it is a question of meals, surely that might be arranged as in ordinary hospitals. As it now stands, the nurses are naturally very much exhausted when they begin their Sunday night duty, and unless the regulation about afternoon rest be altered the strain must inevitably tell on them.

I have trespassed too long on your patience, but the knowledge that your interest and sympathy are always readily accorded to problems of this kind has led me to trouble you with so many details.

### Christmas Entertainments.

KING'S COLLEGE HOSPITAL.

THE festivities commenced on Christmas Day morning, when each patient found at the bedside a gift of useful articles, warm winter clothing, &c., with toys and crackers for the children. On Christmas Day the dinner consisted of turkey, plum pudding, &c. After tea the men were provided with pipes and tobacco. The annual entertainment was given on Wednesday, the 27th, when the resident medical officers, assisted by many musical friends, gave a series of entertainments in the illuminated and decorated wards. Mr. John Coates, Mr. A. Grover, Mr. Charles Loder, Professor Watson Cheyne (who is just starting for South Africa), and others cheered the patients with their songs. Miss Estelle Marsh (violinist), Miss Dunn-Gardner (harpist), and Mr. Piffard (banjoist) delighted them with many excellent selections. Mr. Van Vleet and Mr. Holder were accompanists. The entertainments were highly appreciated, and in the bright surroundings many a patient's sadness gave way to gladness. Miss Dunn-Gardner most kindly played the harp at the five o'clock evening service in the hospital chapel on Christmas Day. On Thursday evening the children's ward was illuminated. A special tea with cakes and sweets was provided, and a large Christmas tree loaded with toys was also illuminated. The toys were distributed to the children, and the medical officers gave them an amusing entertainment.

ST. MARY'S HOSPITAL.

CHRISTMAS was ushered in at St. Mary's Hospital by the singing of several carols under the dome in the entrance hall on Christmas Eve at half-past eight p.m. The choir consisted of nurses and students, conducted by Mr. Sall, himself a student, who spared no pains to make the enterprise a success. The patients were delighted. A dear old lady in the ophthalmic ward said, "Well, my dear, them carols were just beautiful; but you did not give us enough, that you didn't. I wanted to hear more uf 'em." On Christmas morning every patient received a present—the men, shirts, &c.,; the women, various articles of underwear; and the children, all kinds of surprises. The patients' dinner was served at twelve o'clock p.m., and consisted of turkey and plum pudding (for all allowed to have it). The men were supplied with pipes and tobacco, and they surrounded themselves with a halo of smoke all day long. The wards were tastefully decorated with flags, Chinese lanterns, and plenty of holly and ivy. The Manvers Ward, in particular, had a large motto over the windows, "Peace and Prosperity to England," which struck the eye at once. The patients in this ward have worked hard lately, knitting socks, mufflers, and Tam o' Shanters to send to Lady White for our soldiers in South Africa; and here also a few weeks ago thirty guineas were collected for the widows and orphans of the men who have lost their lives fighting so bravely for us all. The patients had their friends to visit them in the afternoon, and then came off the great event of the day—the ward tea—which commenced all round about half-past four p.m. Wives had husbands to tea; and husbands, wives. They had cups and saucers instead of the uniform mug, and just as much cake as they could eat. In fact there was plenty of everything—no lack of cakes, biscuits, crackers, sweets, apples and oranges, &c. Nurses and students played and sang and did all in their power to amuse the patients. Everything was over by nine o'clock, and all were of one voice in declaring that it had been a really happy Christmas Day.

The annual Christmas tree was held in the board-room on the afternoon of Thursday, December 28th. The children looked very pretty in their bright pink jackets and white pinafores; they were all comfortably arranged on new mattresses, covered with snow-white blankets, in front of the stage. There was a start with Punch and Judy, and then came refreshments—stacks of sponge cakes and mugs of milk for the youngsters, tea hot and strong and all kinds of delicious cakes for the adults. Several students acted as stewards, and took care that everyone had plenty. A magic lantern amused the little people for half an hour, and this was followed by Father Christmas (one of the house physicians dressed in character). The tree, laden with toys, was lighted by electric light, and the effect was very good. Not only did all the children in the hospital get toys, but many who had been patients came as visitors, and went home richer than they came. Amongst the toys sent for the children were several boxes of shells picked up on the beach at Osborne by little Prince Edward of York, and a beautiful doll dressed as a page from *Truth.*

THE CANCER HOSPITAL.

A VERY pleasant Christmas Day was spent by the inmates of the Cancer Hospital, Brompton. It began with carols sung by the nurses before the duties of the day commenced. A short and bright service was held after breakfast, then a substantial dinner was provided. It consisted of turkey, pudding, sweets, fruit, and beer where advisable. In the afternoon each patient was allowed two visitors, tea being provided for them. This permission was much appreciated, for it allowed the union of families so characteristic of the season. Each ward vied in making the decorations and laying out of the tea table the best in the institution. Ferns, crinkled paper, and fairy lamps played a large part in the plan of ornamentation. One ward had relied to a large extent on Liberty art muslin, whilst another, to be in keeping with the present strong patriotic spirit, had kept rigidly to red, white, and blue, even the flowers on the table being in unison. The day closed with singing a few hymns, an American organ and piano being placed in suitable positions, so that each commanded one half of the building. The patients have another treat in store, of which the date is yet unfixed. Two ladies, old and valued friends, e rly in the year invit3 all the patients to tea, when each receives a souvenir of the occasion. One could hardly believe, the Matron says, how treasured these little gifts are.

St. Bartholomew's Hospital.

As early as five o'clock on Christmas morning there was an air of suppressed excitement throughout the hospital. In one of the wards, the two night nurses, who had completed the preparations for Christmas left unfinished the day before, held whispered consultations as to the best time for the distribution of presents. Finally they decided to give them round immediately before breakfast. This was done, and as six o'clock struck the last mug of tea and a mysterious brown paper parcel, all knobs, were simultaneously put on No. 20's locker before turning up the gas. Few required waking. No. 3, generally the hardest to convince it was really breakfast time, was knocking the top off his egg with one of his presents, a pipe decorated with tricoloured ribbon, and beside him lay the others, a pouch of tobacco, two warm vests, and a Christmas card from the chaplain. Loud cries of delight came from the cot where Mattie, a little boy of three and a half years, had found a monkey on a stick staring him in the face, and who was violently blowing the back of a watch, vainly imagining it would thus disclose its works. At seven o'clock the day nurses appeared, bringing with them presents for their fellow workers. The night nurses departed for their Christmas dinner at half-past eight, after which they retired to bed so that they might get up at four p.m. to help entertain the patients and pay visits to the other wards for tea. Many and various were the questions asked by the patients during the day, and their spirits rose higher and higher as the dinner-time drew nearer. "Daddy five won't eat any lunch, nurse!" said the probationer. "He's all right," replied nine, a cheeky boy of ten, "he's only leaving room for dinner." "How many helpings of pudding shall I have, nurse?" this last from a convalescent typhoid. The house physician paid his morning visit and prescribed house physic p.v.n. on a good many boards, leaving the ward as the dinner arrived. Carving for twenty people is generally an arduous task, and for twenty sick people it is doubly hard, but sister tackled the roast beef bravely, and plate after plate disappeared into the ward from the kitchen where it was served. Then came the pudding! This *chef d'œuvre* was triumphantly carried round the ward, crowned with holly, and a little heart-boy was overheard to say, "I should like some of that pudden.' Poor thing; he had to put up with custard and a promise of sponge cake for tea. Dinner was at length finished, and the ward straightened for the visitors—not the patients' relatives, they came on Sunday afternoon. The patients who are well enough got up and their beds were made, after which the crumbs were swept up and the tables arranged up the middle of the ward for a grand display of cakes and sweets. The visitors began to arrive at a little after four to find the patients well in the middle of their repast, and enjoying their tea or coffee in real cups and saucers—not the usual mugs—with thin bread and butter, brown or white, as well as heaps of cake. The visiting physician appeared with his wife, and the bigwigs had tea in sister's room. Before a surplus of cake could have time to pall, the lucky bag went round, and shortly afterwards a conjurer and ventriloquist arrived; this kept the patients thoroughly amused until bed-time. The wards are usually closed to visitors at seven o'clock, and the patients scrambled into bed with temperatures considerably raised above normal, but feeling satisfied that they had enjoyed themselves thoroughly, and could not possibly have eaten anything more.

Hospital for Sick Children, Great Ormond Street.

An entertainment for the out-patients of the Hospital for Sick Children was given on Wednesday, December 27th. It commenced with tea, which was partaken of by 250 children. This was followed by a variety entertainment, including Punch and Judy, and at the close of the performance each child received a parcel containing clothing from the stage. Before they left a toy from the Christmas tree, which was illuminated, was also presented to each. On Friday, the 29th, the in-patients had their treat from three to six. There was a tree in each ward, decked with toys for the children, including a *Truth* toy and one from the Corney Grain Fund. The wards were lighted with fairy lights, and decorated with plants and flowers. A number of visitors interested in the hospital were invited, and the entertainment was greatly enjoyed by all.

Brompton Consumption Hospital.

On Thursday, December 28th, the patients of the Hospital for Diseases of the Chest, Brompton, enjoyed their usual Christmas entertainment. The chief attraction was an enormous tree heavily laden with all manner of pretty things, whilst round the foot were a large number of parcels. At six o'clock every patient who could possibly manage to be present assembled in the hall, which was gaily decorated. Those who could not walk were carried in and accorded the best places, whilst the interests of those who were too ill to leave their own quarters were carefully guarded by their nurses. Everyone of the 320 in-patients received something from that wonderful tree. Friends kindly filled up the interludes with music, and a Punch and Judy show afforded a fund of unfailing amusement. This hospital has a great number of friends, and the materials for the Christmas festivities have been liberally provided. This is as it should be, for the nurses, who all work so hard to make the season a happy one, often severely tax their own resources rather than permit any disappointment to fall upon the patients.

The East London Hospital for Children.

The wards of the East London Hospital for Children, at Shadwell, were transformed into a veritable fairyland for Christmas tide. The children themselves were the elves. How attractive and winsome many of them are is never known until they come under the nurse's skilful hands. More wonderful and beautiful still is the unfailing tenderness and care lavished by nurses on the little ones. "But," say the nurses, "they are such dear sweet mites." They demand love, and what is more, they get it. For instance, Kitty, a young lady of three, when she feels a little sorry for herself, remarks sadly, "No one loves Kitty!" which, of course, at once provokes outward demonstrations from a nurse or sister near at hand. The wards were adorned with spring flowers and evergreens, and the walls and windows were made bright with pictures. The grand festival was held on December 28th from three to six, when a large Christmas tree was despoiled of its treasures, from which all received a gift. An excellent band of the Horse Guards discoursed sweet music, and Punch and Judy delighted an enthusiastic audience. A circumstance which contributed not a little to the general movement was the presence of an unusually large number of convalescents, a large batch of whom were despatched to the convalescent home at Bognor on the following day.

Chelsea Hospital for Women.

The Ladies' Committee of the Chelsea Hospital for Women are very generous towards the nurses and inmates at Christmas time. The matron received a lovely box of lilies of the valley, and each nurse a book as gifts, whilst Christmas fare was liberally provided for all. The nurses took special pride in decorating their respective wards, and efforts to carry out systematic schemes of colour were very successful. For example, "Alexandra" was patriotic and clung to the Red,

White, and Blue; "Albert Edward" was adorned with snow and evergreens, whilst on the Albany floor one ward was decorated in white and yellow, and another was most effective in blue. The nurses opened the day's proceedings by singing carols, and dinner was served to the patients at midday. In the afternoon tea was provided by the Ladies' Committee, to which each patient was allowed to invite a guest. In the evening the nurses dined with the matron, when the table and appointments were made as festive as possible. On the afternoon of December 30th the nurses had a private concert of their own, to which they invited the matron. Miss Florence Christie, assisted by some professional friends and a large number of the nursing staff, rendered a varied programme most successfully. Miss Christie kindly visited for some weeks previously the hospital and coached the nurses. Nurse Cleaver acted as accompanist, whilst Nurse French and Nurse Slee each sang solos.

### Great Northern Hospital.

Christmas festivities at the Great Northern Central Hospital took the form of very handsome decorations in the wards, each one being a different scheme of colour, and profusely ornamented with fairy lights and Chinese lanterns, the effect at night being extremely pretty. The patients, hardly without exception, partook of an excellent dinner of roast turkey and sausages, followed by plum pudding and a plentiful supply of such fruit and dessert as the doctors would allow. In the male wards everyone was made supremely happy by being given permission to smoke, and a member of the Ladies' Association having provided each patient with a packet of tobacco, the means were readily at hand. In the afternoon the nursing staff partook of an excellent Christmas dinner in the board-room, and passed an enjoyable hour away from their charges, the latter being looked after in the meantime by the house doctors and the clinical assistants. On Boxing Day several members of the Ladies' Association attended in the afternoon and distributed to every patient useful gifts of clothing and tea in half-pound packets. A party of carol singers, consisting of nurses and friends of the hospital, then visited the wards and entertained the patients with a few carols and songs. This was followed by a tea in each ward, at which everyone had an ample supply of good things provided by the ladies.

### Royal London Ophthalmic Hospital.

The Christmas entertainment of this hospital took place on Thursday, December 28th, in the out-patient department. Nearly all the in-patients were able to come downstairs, and a large number of visitors and friends of the hospital were present. The programme, which was entirely carried out by members of the London Hospital, was as follows: "Mrs. Jarley's Waxworks," "The Old Crocks," farce, "Chiselling," "The Naval Brigade." Refreshments were served in the boardroom. The general arrangements were under the supervision of Miss Richards, who has lately been appointed matron. A vote of thanks to the members of the London Hospital who had so generously given their services on this occasion brought a most pleasant entertainment to an end.

### University College Hospital.

The annual entertainment for the patients in this hospital took place on Friday last, and was much enjoyed. The corridors were prettily decorated and illuminated, and the wards retained much of the festive look they wore at Christmas. Those patients who were able enjoyed an excellent miscellaneous entertainment in the out-patients' department, whilst those who were confined to bed were amused by Punch and Judy, ventriloquists and conjurors, and other shows. The entertainment being held on visiting day the building for some time was rather crowded.

### The East End Mothers' Home.

The usual festivities were held on Boxing Day at the East End Mothers' Home—"Our Home," as the poor mothers of that neighbourhood love to call it. The convalescent patients and the husbands of all the in-patients—who had been invited to tea at five o'clock—assembled and witnessed four pretty tableaux representing the "Seasons," with a fifth introducing Santa Claus in person. A well-decked Christmas tree was subsequently despoiled, and everyone made happy by receiving a gift. Most of the parcels were tied up in coloured wrappers and placed on a little table in readiness for distribution. After this three nurses, dressed in character, sang "Three Old Maids of Lee," which caused the greatest amusement. Carol singing made an appropriate ending to what the patients said was the most enjoyable evening they had ever spent. On Saturday the tree was again lighted and decked for the benefit of some thirty out-patients, who were entertained to supper. These festivities have been provided entirely by friends, many of whom are old pupils.

### West Ham Hospital.

The wards of West Ham Hospital were very prettily decorated for the Christmas merrymaking. Curtains of coloured muslin gave a bright and cosy appearance, whilst moss, flowers, and plants were daintily arranged. On Saturday the authorities held an "At Home" to witness the disrobing of a splendid tree in the children's ward. There were toys and sweetmeats for the children, suitable articles of warm clothing for the men and women, and good fare and kindly greetings for all. Archdeacon Stevens bade the guests welcome in a few well-chosen words, and referred to the loss sustained by the hospital through the death of the Duke of Westminster. The committee have been much gratified by receiving a grant from the Prince of Wales's Hospital Fund of £520, and the promise of an annual subscription of £300 from the same source.

### Mill Lane Hospital, Liscard.

Christmas Day opened very brightly and joyfully for everybody at Mill Lane Hospital, Liscard. In the morning the matron was presented by her nurses with a copper and brass afternoon tea kettle and lamp. During the evening presents were distributed to the patients from the Christmas tree by the matron and charge nurse, all joining in games. On Wednesday, the 27th, the nurses and their friends enjoyed thoroughly a dance given by the matron at the hospital with the sanction of the medical officer and committee. The presents for the tree were kindly sent by the chairman and committee, the attending medical staff, and numerous friends. On January 10th several of the nurses will attend a military ball in aid of the War Fund.

### Royal West of England Sanatorium, Weston-super-Mare.

The annual Christmas tea and concert at the Royal Sanatorium, Weston-super-Mare, were given on Saturday, the 23rd. The lady superintendent and her nurses presided at the tables. The invited guests appeared to enjoy themselves equally with the patients. The tea tables were quite fairy-like, with numerous wax candles in Japanese stands. Sandwiches, cakes in profusion, and sweet dishes of all kinds were greatly enjoyed. Each one had a box of chocolate, with a programme of the concert to follow. The large and lofty reading-room was decorated for the concert. An admirable programme was gone through, several of the nurses taking part in it.

### OUR CLOTHING DISTRIBUTION.

We acknowledge with thanks the receipt of a parcel of five articles from "Nurse Mary."

### Echoes from the Outside World.

AN OPEN LETTER TO A HOSPITAL NURSE.

"A Happy New Year to you all." Such words flow naturally from my pen, writing the first letter in 1900, though the present shadow, which hides the Sun of Peace, tinges even the time-honoured wish with sadness. But a year does not consist only in the few opening weeks, and we may at least hope that before the last twelve months of a dying century have passed away we may be once more enjoying the blessings of former years. The most grievous reflection is that we cannot, alas ! recall those whom we have lost, and that the stream of bloodshed and sorrow must burst out afresh before we can expect a cessation of hostilities. Those who are now enrolling themselves in Durban for the new Railway Pioneer Corps are enlisting for a year's service, which seems to inditnte the opinion prevalent in Natal as to the probable duration of the war. But though the end may be difficult to see, it is a great matter for thankfulness that it is equally difficult to see the end of our resources. The Indian Princes are coming forward with offers of help. The Maharajah of Gwalior has asked to serve at the Cape, and volunteers to send troops, transport, and horses. The Nizam of Hyderabad says that his sword, his army, and his purse are at the services of her Majesty, and every native Indian chief expresset a desire to forward steeds. Whether these offers will be accepted or not is, of course, another matter. A friend in British Columbia writes to me that the keenest disappointment was felt at so few of their men being wanted for the Canadian contingent, and that three or four times the number originally required are eager to go. In less than ten days after they were accepted by the Imperial Government the New South Wales battery set sail for South Africa ; the Victorian corps is forming fast ; Ceylon is equipping 125 men and horses ; New Zealand will send her second contribution a fortnight hence, with more to follow ; and Nova Scotia wishes to assist with a special contingent of its own. We have, indeed, much to be thankful for, and our reverses will have taught us many lessons which could only be learnt on the spot. As someone has pointed out, if we ourselves have not done all that we had hoped to accomplish, the Boers have failed yet more completely in carrying out their programme. They were to have eaten fish in Durban, and prevented the Army Corps from landing at all. It is admitted on all hands that if they had tried to do so before our men got out, their prophecy might have been verified, but that terror at least belongs to the past.

Such war news as there is to record up to Wednesday, though scanty, is good of its kind. General French, with small loss of life, has driven about 6,000 Boers from the neighbourhood of Colesberg, so that he can occupy the town ; the Queenslanders, under Colonel Pilcher, having made a forced march of twenty-two miles round the position, suddenly surprised the Boers at Sunnyside, and captured their laager, with forty prisoners ; and the Colonial troops have not lost the position at Dordrecht which they captured last week. These little items of information are very welcome, and help to cheer both the troops in Africa and us at home ; but it is ridiculous to speak of any of the engagements as a "great victory," or a "splendid success in Natal." Matters are still as serious as ever. Sir George White, in Ladysmith, speaks of fever and dysentery so much on the increase that civilians have been asked to help nurse in the hospital ; Lord Methuen has to remain within his own entrenchments ; Sir Redvers Buller has not crossed the Tugela ; and Kimberley and Mafeking are no nearer being relieved. But help is coming surely, though slowly, and it is consoling to note that the last three successes, achieved alike by our own troops, Queenslanders, and Cape Mounted Rifles, have been won by judicious use of cover, in Boer-like style. So at last our men seem to be learning the hard lesson that concealment pays better than courage.

Colonel Baden-Powell has already had a great deal to contend with in providing food for the besieged and in making arrangements to husband his little army of defenders to the utmost. But lately he has had his troubles added to by his chivalrous desire to assist Lady Sarah Wilson. You may remember that this lady left Mafeking at the commencement of the siege, and by means of heavy bribes succeeded in persuading natives to carry information to and fro. She photographed the wreck of Nesbit's armoured train, and got herself taken, as the sister of a young Boer, to Vryburg, which she afterwards left with secret despatches. But the Boers discovered the dangerous game she was playing, and General Snyman, treating her as a prisoner of war, refused to allow her to return to Mafeking at all unless a notorious wrong-doer, Viljoen, were given up. For some time Colonel Baden-Powell declined to accede to these terms, but ultimately his chivalry gained the day and the lady of title was exchanged for a horse thief. Whereupon Lady Sarah returned once more to her bomb-proof house in the besieged city, where apparently she sits and dispenses hospitality, whisky and soda and cigarettes, to her visitors. I am afraid that women, with the notable exception of nurses, are sadly in the way at the front, and only give a good deal of extra anxiety to the commanders. It is much to be hoped that the "exciting experiences" of one of our sex will not lead to attempts on the part of others to gain a similar experience.

I visited some of the principal London shops on Monday, notwithstanding the trying fog, and heard complaints on all sides that the weather had played sad havoc with the expected number of visitors. Decidedly, there was an entire absence of the usual bustle and scurry which attend the first day of a sale, and, instead of imploring the shopwalker for an assistant to serve, there were plenty of men and women disengaged at all times. The greatest reduction in prices seemed to be in evening dresses and evening materials, because here, naturally enough, the war has affected the market. The first thing to be cut off by those who want to save money is entertaining, and many ladies who usually are conspicuous as hostesses are in mourning just now, and therefore do not keep open house. I looked around for any sign of additional provision of seats for servers, as by the new Act, which comes into operation with the New Year, it is now imperative that every shop shall have a certain number of resting-places for their employees. The appearance of these little shelves—which generally draw out from beneath the counter and slip back automatically when no longer required—is sufficiently familiar to many who buy in the well-regulated West-end emporiums. But it is in small suburban shops that the relief will be most felt. There the assistants, not being drawn from the best class, have hitherto often had scanty consideration given to their comfort—though, of course, there are many exceptions—and the new Act should at least do something towards remedying the ills which come from constant standing. It was in accordance with the fitness of things that a peerage should have been given to Sir John Lubbock upon the same day as that which brought into force a measure for helping afresh a class which has benefitted enormously by the introduction of Bank Holidays. Assistants in shops and bank clerks have all to gain and nothing to lose by a Bank Holiday, and to these Sir John should be almost the "Saint Lubbock" he is sometimes laughingly called. It is to be hoped that he will follow several recent examples, and not hide a name which has become a household word under a brand-new title.

### The Princess of Wales's War Fund.

We publish to-day a further list of subscriptions from nurses to the Princess of Wales's War Fund. All subscriptions should be addressed to the Manager, Office of The Hospital, 28 and 29, Southampton Street, Strand, London, W.C., and the name and address should always be given. The list will close at the end of the month.

Amount already acknowledged, £120 0s. 3d.

| | s. | d. |
|---|---|---|
| C. Brittlebank, P.F. ... | 5 | 0 |
| Rosa Gibbs, P.F. ... | 4 | 6 |
| Nurse Spooner ... | 4 | 0 |
| H. A. Shewan... ... | 4 | 0 |
| Miss Michie ... ... | 3 | 0 |
| A. Wetherell, P.F. ... | 3 | 0 |
| J. S. Downer, P.F. ... | 3 | 0 |
| S. Annie Allan, P.F.... | 3 | 0 |
| Kate Ashford, P.F. ... | 3 | 0 |
| E. A. Franks, P.F. ... | 2 | 0 |
| A. J. Sinton, P.F. ... | 3 | 0 |
| Mary Crofts, P.F. ... | 3 | 0 |
| Policy 6,423, P.F. ... | 3 | 0 |
| M. S. L. Hillhouse, P.F. | 3 | 0 |
| M. E. Davies, P.F. ... | 2 | 6 |
| A. S. Rhind, P.F. ... | 2 | 6 |
| E. Mackay, P.F. ... | 2 | 6 |
| E. Mayston, P F. ... | 2 | 6 |
| A. Willie, P.F. ... | 2 | 6 |
| Matron, P.F. ... ... | 2 | 6 |
| A. Binnie, P.F. ... | 2 | 6 |
| Policy 1,611, P.F. ... | 2 | 6 |
| J. Boyd, P.F. ... ... | 2 | 6 |
| Nurse Ross, P.F. ... | 2 | 6 |
| H. M. Hill, P.F. ... | 2 | 6 |
| Policy 3,897, P.F. ... | 2 | 6 |
| Matron, P.F. ... ... | 2 | 6 |
| Pauline Schor ... ... | 2 | 6 |
| Ella Wilkin, P.F. ... | 2 | 6 |
| Sister Briant, P.F. ... | 2 | 6 |
| Bessie Fulcher, P.F. ... | 2 | 6 |
| S. Daley, P.F.... ... | 2 | 6 |
| M. P. Dunn ... ... | 2 | 6 |
| Gertrude Somers-James | 2 | 6 |
| M. Donelly, P.F. ... | 2 | 6 |
| A. Solomon, P.F. ... | 2 | 6 |
| A. Marwood, P.F. ... | 2 | 6 |
| C. Andrews, P.F. ... | 2 | 0 |
| Alice E. Ridsdale ... | 2 | 0 |
| Frances E. Mansell, P.F. ... ... ... | 2 | 0 |
| Nurse Priddees, P.F.... | 2 | 0 |
| Miss E. Wollway, P.F. | 2 | 0 |
| Emily Read, P.F. ... | 2 | 0 |
| Kate Smith, P.F. ... | 2 | 0 |
| Nurse Macfarlane, P.F. | 2 | 0 |
| Mary E. Jones, P.F.... | 2 | 0 |
| Emily Brindery, P.F. | 2 | 0 |
| Mary Homer, P.F. ... | 2 | 0 |
| Ellen Smith, P F. ... | 2 | 0 |
| J. E. R., Policy 5,116, P.F. ... ... | 2 | 0 |
| Policy 6,343, P.F. ... | 2 | 0 |
| Eliza E. Cope, P.F. ... | 2 | 0 |
| E. Smith, P.F. ... | 2 | 0 |
| J. Ningeton, P.F. ... | 2 | 0 |
| E. C. Chawner, P.F.... | 2 | 0 |
| Helen Jones, P.F. ... | 2 | 0 |
| M. E. Sutherland, P.F. | 2 | 0 |
| Gertrude E. Male, P.F. | 2 | 0 |
| E. S. Salsbury, P.F.... | 2 | 0 |
| Mary A. Harris, P.F. | 2 | 0 |
| E. J. Daniels, P.F., and Mary Davies, P.F.... | 2 | 0 |
| Nurse Osborne, P.F.... | 2 | 0 |
| Ada Pearson, P.F. ... | 2 | 0 |
| H. C. Spendlove, P.F. | 2 | 0 |
| L. J. Gervis, P.F. ... | 2 | 0 |
| Florence Tudor, P.F.... | 2 | 0 |
| E. Moule, P.F. ... | 2 | 0 |
| M. S. ... ... ... | 2 | 0 |
| H. R. Ellison, P.P. ... | 2 | 0 |
| Emily Dinnie, P.F. ... | 2 | 0 |
| E. Manwaring, P.F., & M. Manwaring, P.F. | 2 | 0 |
| F. E. M. Pike, P.F. ... | 2 | 0 |
| Nurse Bennett, P.F.... | 2 | 0 |
| R. E. Morrow, P.F. ... | 2 | 0 |
| Margaret J. McCartney, P.F. ... ... | 2 | 0 |
| Agnes Gibson, P.F. ... | 1 | 6 |
| Helen Rawlings, P.F. | 1 | 6 |
| Nurse James, P.F. ... | 1 | 6 |
| Amy Hallam ... ... | 1 | 6 |
| Nurse Clare, P.F. ... | 1 | 6 |
| Miss Proschwitzky, P.F. | 1 | 6 |
| S. Watts, P.F. ... | 1 | 6 |
| Sister Hillman, P.F. ... | 1 | 6 |
| J. M. Lysaght, P.F. ... | 1 | 6 |
| H. L. Arnold, P.F. ... | 1 | 0 |
| R. M. MacPhail, P.F. | 1 | 0 |
| J. Ritchie, P F. ... | 1 | 0 |
| B. J. East, P.F. ... | 1 | 0 |
| J. Souter, P.F. ... | 1 | 0 |
| Nurse Clements, P.F. | 1 | 0 |
| Mary McNab, P.F. ... | 1 | 0 |
| Nurse Yeoman, P.F.... | 1 | 0 |
| A. Botwood, P.F. ... | 1 | 0 |
| A. Gloster, P.F. ... | 1 | 0 |
| Mrs. C. S. Clegg, P.F. | 1 | 0 |
| Nurse Cutting, P.F. ... | 1 | 0 |
| E. Croft, P.F.... ... | 1 | 0 |
| L. Benson, P.F. ... | 1 | 0 |
| Policy 6,078 ... ... | 1 | 0 |
| Florence A. Weire, P.F. | 1 | 0 |
| Miss M. Cook, P.F. ... | 5 | 0 |
| Nurse Beere ... ... | 5 | 0 |
| Nurse McLeod... ... | 2 | 6 |
| Nurse Rolleston ... | 2 | 0 |
| Nurse Wright, P.F. ... | 1 | 0 |
| Nurse Taylor ... ... | 1 | 0 |
| Nurse Burgess... ... | 1 | 0 |
| Nurse Joynes ... ... | 1 | 0 |
| Nurse Fitzgerald ... | 1 | 0 |
| Nurse French ... ... | 0 | 6 |
| Mary Shirley, P.F. ... | 5 | 0 |
| Jessie E. Roberts, P.F. | 3 | 0 |
| Katherine Bickerton... | 2 | 0 |
| Nurse Shoesmith, P.F. | 2 | 0 |
| Nurse Barnes, P.F. ... | 2 | 0 |
| Nurse Green, P.F. ... | 2 | 0 |
| E. Howe, P.F. ... | 5 | 0 |
| Nurse Travis ... ... | 2 | 0 |
| E. Lyon, P.F.... ... | 3 | 0 |
| E. Lyon, P.F.... ... | 1 | 0 |
| Mary Ormond, P.F. ... | 5 | 0 |
| Jane Jeffery, P.F. ... | 5 | 0 |
| A. Bond, P.F. ... | 5 | 0 |
| Edith Coutts, P.F. ... | 5 | 0 |
| A. E. Nicholson, P.F. | 5 | 0 |
| M. B. Herd, P.F. ... | 5 | 0 |
| Eleanor Walker, P.F. | 5 | 0 |
| Policy 4,593, P.F. ... | 5 | 0 |
| K. M. Pierce ... ... | 5 | 0 |
| Ethel Lawrence, P.F. | 5 | 0 |
| Mary Gardner, P.F. ... | 5 | 0 |
| Rosa A. Potts, P.F. ... | 5 | 0 |
| Clara Clews, P.F. ... | 3 | 0 |
| Kate Kimber ... ... | 3 | 0 |
| A. H. Waddell, P.F.... | 2 | 6 |
| Helen Dawson, P.F. ... | 2 | 6 |
| A. A. Foulkes, P.F. ... | 2 | 6 |
| H. Kenten, P.F. ... | 2 | 6 |
| Henrietta J. Moore, P.F. | 2 | 6 |
| Katherine Tilt, P.F. ... | 2 | 6 |
| Anne M. Coppin, P.F. | 2 | 6 |
| E. H. Thomson, P F. | 2 | 6 |
| Policy 5,398, P.F. ... | 2 | 6 |
| Laura B. Penison, P.F. | 2 | 6 |
| M. A. Rogers, P.F. ... | 2 | 6 |
| J. Reid, P.F. ... ... | 2 | 6 |
| O. A. Beaumont, P.F. | 2 | 6 |
| J. Heriot, P.F... ... | 2 | 6 |
| Nora C. Grist, P.F. ... | 2 | 0 |
| Catherine Terry, P.F. | 2 | 0 |
| A. W. Walkinshaw, P.F. | 2 | 0 |
| F. S., P.F. ... ... | 2 | 0 |
| Arabella Craydon ... | 2 | 0 |
| Olive Ashley, P.F. ... | 2 | 0 |
| Nurse Pagan, P.F. ... | 2 | 0 |
| Nurse Sanders, P.F. ... | 2 | 0 |
| Jane Macfarlane, P.F. | 2 | 0 |
| Mary A. Baynes, P.F. | 2 | 0 |
| Nurse Harvey, P.F. ... | 2 | 0 |
| J. J. Christie, P.F. ... | 2 | 0 |
| C. Macpherson ... | 2 | 0 |
| Charity Weekes, P.F... | 1 | 6 |
| H. Beachem, P.F. ... | 1 | 6 |
| Amelia Sander, P.F.... | 1 | 0 |
| Eliza North, P.F. ... | 1 | 0 |
| E. F. Tubbs, P.F. ... | 1 | 0 |
| S. H. Stead, P.F. ... | 1 | 0 |
| Policy 2,283, P.F. ... | 1 | 0 |
| J. Pugh, P.F.... ... | 1 | 0 |
| Nurse Balderson ... | 1 | 0 |
| M. Baldwell, P.F. ... | 1 | 0 |
| S. A. C., P.F.... ... | 1 | 0 |
| C. M. Lohr, P.F. ... | 2 | 6 |
| Nurse Jacob ... ... | 1 | 6 |
| Policies 4,572 and 3,381, P.F. ... ... ... | 5 | 0 |
| D. N. Newman, P.F.... | 5 | 0 |
| Mary Wilkinson, P.F. | 5 | 0 |
| Miss H. M. Gooding, P.F. | 5 | 0 |
| Ada Butler, P.F. ... | 5 | 0 |
| M. H. Rosher... ... | 5 | 0 |
| Anna Chapman, P.F.... | 5 | 0 |
| H. Scott, P.F.... ... | 3 | 6 |
| Emily Johns, P.F. ... | 3 | 0 |
| Nurse Wickers, P.F. ... | 3 | 0 |
| T. Harewood, P.F. ... | 3 | 0 |
| Eliza Mary Peter, P F. | 3 | 0 |
| F. Gaylard, P.F. ... | 2 | 6 |
| Eliza Durham, P.F. ... | 2 | 6 |
| Sarah Baxter, P.F. ... | 2 | 6 |
| Nurse Bright ... ... | 2 | 6 |
| E. A. Ankritt, P.F. ... | 2 | 6 |
| A. P. Attenborough ... | 2 | 6 |
| Mary Spratt, P.F. ... | 2 | 6 |
| Nurse Vaughan, P.F... | 2 | 6 |
| J. Thodey, P.F. ... | 2 | 6 |
| L. A. Hyder, P.F. ... | 2 | 6 |
| L. V. Houghton, P.F. | 2 | 6 |
| Miss Hawahan, P.F.... | 2 | 0 |
| Sarah Fox, P.F. ... | 2 | 0 |
| E. Clark, P.F.... ... | 2 | 0 |
| Nurse Settle, P.F. ... | 2 | 0 |
| C. M. Wood ... ... | 2 | 0 |
| R. Milsom, P.F. ... | 2 | 0 |
| Nurse Fenson, P.F. ... | 2 | 0 |
| Miss Farrell, P.F. ... | 2 | 0 |
| Ellen E. Wilson, P.F. | 2 | 0 |
| Nurse Pilton, P.F. ... | 2 | 0 |
| H. L. Saunders, P.F.... | 2 | 0 |
| L. Hinton, P.F. ... | 2 | 0 |
| Mary Brewton, P.F.... | 1 | 0 |
| M. E. Meadows, P.F... | 1 | 0 |
| Annie Thorpe, P.F. ... | 1 | 0 |
| Edith Manchester, P.F. | 1 | 0 |
| Miss Stewart, P.F. ... | 1 | 0 |
| Miss L. A. Taylor, P.F. | 1 | 0 |
| Miss A. D. Marriner, P.F. ... ... ... | 5 | 0 |
| Nurse McLaren ... | 3 | 0 |
| Nurse Mapstone ... | 2 | 0 |
| Nurse Lamb ... ... | 1 | 0 |
| Alice Taylor ... ... | 1 | 0 |
| Emily C. Hope, P.F.... | 5 | 0 |
| A. Gough, P.F. ... | 5 | 0 |
| M. Gough, P.F. ... | 5 | 0 |
| Miss F. Nicholson ... | 5 | 0 |
| Lizzie E. Clifton, P.F. | 5 | 0 |
| E. J. Munsey ... ... | 5 | 0 |
| Edith A. Hull, P.F. ... | 5 | 0 |
| Lilian E. Cope ... | 5 | 0 |
| Mary B. Fennell, P.F. | 5 | 0 |
| Nurse Worsey, P.F. ... | 5 | 0 |
| Charlotte Collings, P.F. | 3 | 0 |
| Nurse Hudson, P.F. ... | 3 | 0 |
| Isabel H. Hughes, P.F. | 3 | 0 |
| B. D. Policy 4,254, P.F. | 2 | 6 |
| L. E. P. Policy 6,703, P.F. ... ... ... | 2 | 6 |
| Mary D. Watson, P.F. | 2 | 6 |
| Mary A. Gibbs, P.F. ... | 2 | 6 |
| H. Peck, P.F. ... ... | 2 | 6 |
| E. G. Barrett, P.F. ... | 2 | 6 |
| Mary Williams, P.F. ... | 2 | 6 |
| L. Hotson ... ... | 2 | 6 |
| Isabel Rylands, P.F. ... | 2 | 6 |
| Agnes Jesse, P.F. ... | 2 | 6 |
| C. Baker, P.F. ... | 2 | 6 |
| Fanny G. Davis ... | 2 | 6 |
| E. J. Cox ... ... | 2 | 6 |
| Nurse Crowsley, P.F. | 2 | 6 |
| Winifred Phillips ... | 2 | 0 |
| S. Griggs, P.F. ... | 2 | 0 |
| Lucy Colbeck, P.F. ... | 2 | 0 |
| Policy 389, P.F. ... | 2 | 0 |
| M. E. Keown, P.F. ... | 2 | 0 |
| Amy Jane Bullock, P.F. | 2 | 0 |
| Edith J. Hemus, P.F. | 2 | 0 |
| Eliza J. Lander, P.F. | 2 | 0 |
| L. M. Griffin, P.F. ... | 2 | 0 |

Collected at the Nurses' Tea Party, 27, Brompton Square, December 19th, 1899.

| | s. | d. |
|---|---|---|
| G. H. C. ... ... | 5 | 0 |
| G. Falkiner ... ... | 1 | 0 |
| E. W. R. ... ... | 2 | 6 |
| Margaret Hendrie ... | 2 | 6 |
| F. M. Marword ... | 2 | 0 |
| Edith W. Kersey ... | 2 | 0 |
| Eileen O'Donovan ... | 2 | 6 |
| Arthur Fitzgerald ... | 2 | 6 |
| Norah Wilkinson ... | 2 | 6 |
| Marjory MacDougall ... | 2 | 6 |
| Elvira Coates ... ... | 2 | 6 |
| Annie Harsant ... | 4 | 0 |
| Emily Chaplin ... | 2 | 0 |
| Adela C. Villiers ... | 5 | 0 |
| Mrs. Mulhalt ... ... | 2 | 0 |
| H. Richards ... ... | 1 | 6 |
| A. Denman ... ... | 2 | 0 |
| Mrs. Antonini ... | 1 | 6 |
| Lilian Sheppard ... | 2 | 6 |
| Mrs. Ambrose ... | 2 | 0 |
| Nurse Mello ... ... | 2 | 6 |
| Isabelle Smith ... | 5 | 0 |
| Elizabeth West ... | 2 | 6 |
| Emily Wardle ... | 1 | 0 |
| Emilie Stone ... ... | 5 | 0 |
| Bedina Hayes ... ... | 2 | 6 |
| Frances Mitchell ... | 1 | 0 |
| Nurse Tarrant ... | 5 | 0 |
| H. A. H. Walker ... | 10 | 0 |

### Everybody's Opinion.

[Correspondence on all subjects is invited, but we cannot in any way be responsible for the opinions expressed by our correspondents. No communication can be entertained if the name and address of the correspondent is not given, as a guarantee of good faith but not necessarily for publication, or unless one side of the paper only is written on.]

### OUR CLOTHING DISTRIBUTION.

The Lady Superintendent of the East End Mother's Home writes: Now that Christmas is over I hasten to thank you for bringing us such a splendid parcel of clothing; we are, indeed, grateful for your kind help.

The Secretary of the East London Hospital for Children and Dispensary for Women, Shadwell, E., writes: I am directed by the Board of Management to convey to you an expression of their sincere thanks for your kind and useful present of clothing, which is much appreciated.

The Matron of the Metropolitan Hospital, Kingsland Road, writes: Thank you so much for the very useful parcel of clothing, &c., which you sent to us for Christmas gifts to the patients. All the garments were most acceptable, and the patients were delighted with them. We are very grateful to you for remembering us.

### THE TRAINING OF NURSES FOR THE WAR.

"E. T." writes: "How is it that in your article on "The Princess of Wales's Nurses for the War" a full account of the training of several nurses is given, while in that of others no details are supplied?"

*** We published all the information we possessed. This inquiry affords us an opportunity of urging all nurses who wish to enjoy the obvious advantages of having their training and past experience stated when they are appointed to posts of importance to send in full details of their career for publication in the next issue of "Burdett's Official Nursing Directory." Unless this information is ready to hand, it is not always practicable to obtain it, but nurses who forward particulars of their past and present work for the "Directory" ensure a correct and full account of it in The Hospital when they are selected for promotion or special honour.

[We are compelled to hold over a number of interesting letters.]

### The Queen's Nurses' Appointments for 1900.

The Queen has approved the appointment of the following nurses to Queen Victoria's Jubilee Institute for Nurses, to date January 1st, 1900:—

England.—Anne Clementina Halliday, Frances Shaw, Annie Tait Cleo Henderson, Annie Louise Bacon, Nellie Gordon Cumming, Adela Eliza Higgins, Amy Frances Noaks, Alice Kathrine Rawlins, London; Marion Mackenzie Bruce, Theresa Margaret Harris, Gertrude Lilly, Alice Annie Cowley, Edith Howell, Martha Isabella Warburton, Liverpool; Annie Jane Youngman, Emma Louisa Johnson, Sarah Lees, Wilhelmina Mathieson, Catherine Meredith, Ada Milner, Jeanie Easton Martin, Caroline Berry, Helen Orr, Manchester; Martha Bishop, Mary Dix, Salford; Sarah Hannah Hodgson, Clara Reeve, Leeds; Jane Emma Taylor, Edith Bentley, Ruth Amy Finlinson, Hull; Annie Ely Mills, Gateshead; Gertrude Haynes, Kate Amelia Clarkson, Helen Augusta Alexander, Brighton; Alice Marian Tilby, Phœbe Florence Chatfield, Gloucester; Christina Jane Aldis, Lottie Ridler, Clara Beatrice Beeley, Ashton-under-Lyne; Henrietta Maria Fischer, Rochdale; Johanna McMillan, Cleator; Alice Barber, Chorlton-cum-Hardy; Emily Helen Rymer, Consett; Annie Lucie Blackburn, Blackburn; Emily Blanche Errington, Liversedge; Clara Rose Page, Heywood; Harriet Emily Bennitt, Pudsey; Jane Cooper Baylee, Buxton; Frances Ellen Towns, Bedford; Harriet Rose Heale, Coventry; Hattie Louisa Philp, Warwick; Helen Augusta Emily Taylor, Faringdon Elizabeth Ada Lee, Bridgwater; Maria Glynn, Kingston-on-Thames Blanche Grace Beatrice Pheil, Hadleigh Ada Frances Waind, Surbiton; Sarah Gilbert, Littlebourne; Esther Jane Pearce, Penshurst; Lucy Marshall, Portsmouth; Annie Maud Martindale, Paignton; Eugènie Widt, Birmingham; Emily Eveline Connell, Peterborough.

Wales.—Mary Burnett, Cardiff; Elizabeth West Thorpe, Pembroke Dock; Mary Anne Johns, Ystalyfera; Sophie Jane Hannon, Jane Elizabeth Read, Aberystwith; Isabella Farren, Llanrwst; Eliza Jane Evans, Trevor; Anita Schönberg, Halkyn; Jane Elizabeth Phillips, Llanerchymedd.

Scotland.—Marianne Menzies Macdonald, Elizabeth Russell, Marjory Macnicoll Low, Mary Wilson, Margaret Munro, Edinburgh; Elizabeth Mitchell McLellan, Isobella Allan McAlister, Agnes Ronald Baird, Flora McMillan, Glasgow; Isabella Boyd, Elizabeth Margaret Thomson, Hawick; Emma Banner, Airdrie; Mary Hayton, Blantyre; Mary Elizabeth Todd, Barrhead; Margaret Mackay, Forth; Jane Cumming Gray Donald, Wick; Marion MacLeod MacDonald, Stornoway; Mary Mackinven, Morven, Argyllshire; Margaret McAfee, Armadale.

Ireland.—Mary Frances Hipwell, Sara Ellen Mathilde McKenna, Rebecca Lillian Kane, Mary Maud Stockwin, Mary Macaulay, Dublin; Elizabeth Reidy, Burriscarra; Mary Anne Shanahan, Swords; Josephine Bird, Oughterard.

### Appointments.

Isolation Hospital, Hinckley.—Miss A. E. Gillespie has been appointed Nurse Matron. She was second assistant nurse for ten months at the North-Eastern Fever Hospital, South Tottenham, and then trained for three years at the Fir Vale Union Infirmary, Sheffield. She has since been charge nurse at Coventry Fever Hospital, and head nurse at Cannock Union Infirmary.

Southend Victoria Hospital.—Miss E. Radburn has been appointed Matron. She was trained at the Nightingale Training School, St. Thomas's Hospital. After nursing for seven years in St. Thomas's Hospital, she was for five years attached to the Nursing Institution, Southend, and she has taken charge of the Victoria Hospital during the temporary absence of the matron.

Isolation Hospital, New Pond Farm, Reigate.—Miss Lucy Sophia Price has been appointed Nurse-Matron. She was trained at the London Hospital, and has since been nurse in succession at the Dutch Convalescent Home, Old Charlton, Kent, Swanage Cottage Hospital, and the "Bush" Hospital, under the Metropolitan Asylums Board.

The Royal Orthopædic Hospital, London.—Miss Lucy Wilson Warnsley has been appointed Matron. She was trained at the London Hospital, where she subsequently held the posts of night sister and sister of the Cotton wards. Before entering the London Miss Warnsley had 2½ years' experience in the Children's Hospital, Birmingham.

Kendal Isolation Hospital.—Miss Edith Haspell has been appointed Matron. She was trained at Kendal Sanatorium, and has since been staff nurse at the City Hospital, Liverpool, and charge nurse and assistant matron at Blawarthill Hospital, Yoker, near Glasgow.

Allt-yr-yn Fever Hospital, Newport, Mon.—The name of the new matron is Greenlaw, not Greenland.

### Minor Appointments.

Birmingham General Hospital.—Miss M. J. Martin has been appointed Ward Sister. She was trained at the Middlesex Hospital, and has since been sister of the women's surgical wards at Cardiff Infirmary for two years.

### TRAVEL NOTES AND QUERIES.

Mentone (Fairhazel).—Why do you leave your queries till the last moment? The home you speak of is charitable. I should try the Hotel Pension Santa Maria. Pension from 7½ to 12 francs; excellent situation. Mentone is one of the most charming situations on the Riviera, and not so much afflicted with the mistral as some. This is a north-west wind of extreme violence, almost a tornado at times. I do not advise Hyères if your friend is much tied to the house. S. Raphael is very quiet. I should prefer Mentone myself. You can see what you wish in Paris in one day, but it would be very fatiguing, and unadvisable except for the robust.

### For Reading to the Sick.

### THE EPIPHANY.

AND, lo, the star, which they saw in the east, went before them, till it came and stood over where the young child was. When they saw the star, they rejoiced with exceeding great joy.—*St. Matthew* ii. 9, 10.

Star of the East, how sweet art Thou,
Seen in life's early morning sky,
Ere yet a cloud has dimmed the brow,
While yet we gaze with childish eye.
—*Keble.*

Oh, let our adoration for all that He hath done
Peal out beyond the stars of God, while voice and life are one!
And let our consecration be real, and deep, and true,
Oh, even now our hearts shall bow, and joyful vows renew!

In full and glad surrender we give ourselves to Thee,
Thine utterly, and only, and evermore to be!
O Son of God, Who lovest us, we will be Thine alone,
And all we are, and all we have, shall henceforth be Thine own! *F. R. Havergal.*

Bring thine all, thy choicest treasure,
Heap it high and hide it deep!
Thou shalt win o'erflowing measure,
Thou shalt climb where skies are steep.

For as Heaven's true only light
Quickens all those forms so bright,
So where Bounty never faints
There the Lord is with His saints. —*Keble.*

**Reading.**

What joy the sages conceived when their eyes first beheld the reappearance of that happy Star, they only can tell who, after a long and sad night of temptation, have seen the loving countenance of God shining forth upon their souls. If, with obedience and courage, we can follow the calling of God in difficult enterprises, we shall not want supplies of comfort. Let us not be wanting to God; we shall be sure He cannot be wanting to us.

He that led Israel by a pillar of fire into the land of promise, leads the Wise Men by a Star to the promised Seed. All His directions partake of that light, which is in Him; God is light.—1 *John* i. 5.

"They fell down and worshipped Him."

They which saw His Star afar off in the East, when He lay cradled in Bethlehem, do also see His royalty further off in the despised estate of His infancy; a royalty more than human. They well knew that stars did not use to attend earthly kings; and if their aim had not been higher, what was a Jewish king to Persian strangers?

Neither did they lift up heavy hands to Him whom they worshipped; but presented Him with the most precious commodities of their country, gold, and frankincense, and myrrh; not as thinking to enrich him with these, but by way of homage acknowledging him the Lord of these things. As God loves not empty hands, so He measures fulness by the affection. Let it be gold, or frankincense, or myrrh that we offer Him, it cannot but please Him who doth not use to ask how much, but how good.—*Bishop Hall.*

With bowed heads and open hearts, may we offer ourselves. We can do no more, and we dare do no less.—*Westcott.*

### To Nurses.

IN order to increase and vary the interest in the *Mirror*, we invite contributions from any of our readers in the form of either an article, a paragraph, or information, and will pay a minimum of 5s. for each contribution. All rejected manuscripts are returned in due course, and all payments for manuscripts used are made at the beginning of each quarter, *i.e.*, January 1st, April 1st, July 1st, and October 1st.

### Notes and Queries.

**The contents of the Editor's Letter-box have now reached such unwieldy proportions that it has become necessary to establish a hard and fast rule regarding Answers to Correspondents. In future, all questions requiring replies will continue to be answered in this column without any fee. If an answer is required by letter, a fee of half-a-crown must be enclosed with the note containing the enquiry. We are always pleased to help our numerous correspondents to the fullest extent, and we can trust them to sympathise in the overwhelming amount of writing which makes the new rules a necessity.**

**Every communication must be accompanied by the writer's name and address, otherwise it will receive no attention.**

*Homes of Rest.*

(145) In the *Daily Graphic* for December 16th Lord Pirbright is mentioned as having received a cheque for the Princess Christian Homes of Rest for Disabled Soldiers and Sailors from the Lord Mayor of London from the Mansion House War Funds. To whom should I write to ascertain some more particulars about the Homes of Rest?—*Sister Eva.*

The Secretary, Soldiers and Sailors' Help Society, 17, Shaftesbury Avenue, W. This society is acting in conjunction with the military authorities.

*Nauheim Baths.*

(146) Some time ago I saw in your valuable paper an article on the Nauheim treatment, but cannot remember if the address of the agents that supply the preparation for the baths was given. Will you kindly give me it?—*Bad Nauheim.*

You could probably obtain it by sending a line to the Secretary, Nurses' Co-operation, 8, New Cavendish Street, W.

*Cancer.*

(147) Can you tell me if there is a home in the West of England, Cornwall or Devon preferred, where an old man of 80 suffering with cancer, who has seen better days but has no one to take care of him, could go to end his days? A small sum would be paid for his maintenance.—*A. M.*

St. Barnabas Home for Incurables, Brockett Hall, Torquay, seems suitable for the case. Apply the Sister Superior, St. Raphael's Home, Higher Lincombe Road, Torquay.

*Chronic Catarrh.*

(148) Will you please tell me of some suitable remedy for catarrh in the head? The patient frequently seems either crying or as though she had a bad attack of the old-fashioned influenza. I cannot persuade her to see a doctor, as she once heard one remark that he did not think there was any cure for it.—*Catarrh.*

We can only recommend you to persuade the patient to consult a medical man.

*Private Nursing Homes.*

(149) A lady friend who wishes to join the nursing profession wrote in answer to an advertisement in your paper and received back a form and the enclosed letter, both of which I enclose. I should very much like your opinion *re* the same.—*Knowledge.*

Your friend would be ill-advised to learn nursing in a private home. If she invests 2s. in a copy of the "Nursing Profession: How and Where to Train," she will have a complete list of the recognised training schools in the United Kingdom, and can apply direct to the matrons of such as suit her. It is of the utmost importance to a nurse that her certificate should be from a recognised training school, and for three years.

*Anti-Vivisection.*

(150) Will you kindly tell me what hospitals, &c., practise vivisection? Being a lover of animals, and strongly condemning such practices, I do not wish to give to or work in any such institutions.—*Hull Nurse.*

According to the English law experiments on animals can only be performed under special licence, which licence is never granted to hospitals, but only to individuals.

*Mentally Afflicted.*

(151) Will you please say if you know of a home (near Leeds preferably) where a lady (a governess mentally afflicted through overwork) can be taken for a guinea and a-half a week? Would like her to be admitted soon.—*Nurse F. D.*

It might be worth while to advertise in the Leeds papers.

*Home Remedies.*

(152) Will you kindly tell me what would be a suitable book for a young married lady living quite in the country? Simple treatment and medicine for both adults and children in simple ailments is what is needed.—*District Nurse.*

"Chevasse's Advice to a Mother on the Management of her Children" (J. and A. Churchill, 2s. 6d.) would probably answer the purpose satisfactorily.

*Splint.*

(153) "A. G. W." asks if there could be a splint or case made to keep one knee in its proper position, as her patient's right leg has been so mismanaged that it prevents his walking. Instead of one knee bending as it should it goes backwards. He can manage to stand, but not walk on it.

"A. G. W." should persuade her patient to consult a surgeon as soon as possible.

*Standard Books of Reference.*

"The Nursing Profession: How and Where to Train." 2s. net.
"The Nurses' Dictionary of Medical Terms." 2s. 6d. net.
"Burdett's Series of Nursing Text-Books." 1s. each.
"A Handbook for Nurses." (Illustrated.) 5s.
"Nursing: Its Theory and Practice." New Edition. 3s. 6d.
"Helps in Sickness and to Health." Fifteenth Thousand. 5s.
All published by THE SCIENTIFIC PRESS, Ltd., and may be obtained through any bookseller or direct from the publishers, 28 & 29, Southampton Street, London, W.C.

